# Statistical Analysis of Silicon Photomultiplier Output Signals

**DOI:** 10.3390/s22239134

**Published:** 2022-11-24

**Authors:** Zdenek Kolka, Peter Barcik, Viera Biolkova

**Affiliations:** Department of Radio Electronics, Faculty of Electrical Engineering and Communication, Brno University of Technology, Technicka 12, 616 00 Brno, Czech Republic

**Keywords:** silicon photomultiplier, non-stationary Poisson process, correlated averaging, range finding

## Abstract

Silicon photomultipliers are relatively new devices designed as a matrix of single-photon avalanche detectors, which have become popular for their miniature dimensions and low operating voltage. Their superior sensitivity allows detecting low-photon-count optical pulses, e.g., in ranging and LIDAR applications. The output signal of the photomultiplier is a non-stationary stochastic process, from which a weak periodic pulse can be extracted by means of statistical processing. Using the double-exponential approximation of output avalanche pulses the paper presents a simple analytical solution to the mean and variance of the stochastic process. It is shown that even for an ideal square optical pulse the rising edge of the statistically detected signal is longer than the edge of individual avalanche pulses. The knowledge of the detected waveform can be used to design an optimum laser pulse waveform or algorithms for estimating the time of arrival. The experimental section demonstrates the proposed procedure.

## 1. Introduction

The detection of weak optical pulses has traditionally been based on vacuum photomultiplier tubes or avalanche photodiodes (APD). Photomultipliers are mature and bulky devices with more than 80 years of history. A semiconductor photodiode represents a more practical device that can be miniaturized and easily integrated into an opto-electrical setup. The ultimate goal of sensitivity, i.e., single-photon detection, can be achieved by means of photodiode design modification. APDs working as single-photon avalanche detectors (SPAD) appeared in the 1980s [1,2]. SPAD is a photodiode designed for operation above the reverse breakdown. An incident photon triggers a self-sustaining avalanche, which should be quenched by an external circuitry. The simplest method, known as passive quenching, uses a series resistor. When the avalanche develops, the diode voltage decreases below the breakdown and the avalanche ceases, which creates an easily detectable electrical pulse. However, the pulse waveform does not depend on the number of photons that hit the diode active area.

Silicon photomultipliers (SiPMs) are devices that have appeared only recently [3]. The analog SiPM, which the study is focused on, is a matrix of hundreds of SPADs with integrated quenching resistors, all of them connected in parallel. In case of digital SiPM, the active quenching and detection logic are integrated with each pixel [4]. The size of individual SPAD pixels ranges from 10 μm to 100 μm. The devices are manufactured either by a custom process with vertical photodiode-like current flow or by a CMOS-compatible process with planar structures on a common substrate, which differ in the transient behavior [5]. In contrast to the single SPAD, the current of analog SiPM is in a certain range proportional to the number of detected photons because there are many pixels ready to be triggered [6]. In addition to their linearity, the main advantage of SiPMs is the low operating voltage (tens of volts), which greatly simplifies the design of battery-powered devices, high gain (up to several millions), and insensitivity to magnetic field.

One of the problems of SiPMs is the long tail of output pulses, which may reach up to hundreds of nanoseconds. If their rate is low, they can be easily separated. When the rate grows, the tails start to overlap and the separation of individual pulses is more complex or even impossible. One of the options to filter-out the pulse tail is to couple each SPAD pixel to the output with a capacitor [7]. The high-pass filter created passes only the fast edge of the avalanche pulse. Such devices have been developed by the SensL Company (now ON Semi) and are used in this study.

SiPMs have become popular in applications that require the detection of optical signals with a low photon count. The applications include ranging and remote sensing [8,9], spectroscopy in biology and nuclear sciences [10], quantum physics [11], and numerous other fields [3]. A considerable effort has been devoted to the development of SiPM theory. Similar to APD, the high gain of SiPM is accompanied by excess noise, but the physical essence is different. The avalanche breakdown of one particular pixel may trigger, with a certain probability, one or more neighboring pixels. The physical mechanism includes a direct optical crosstalk with almost no delay or a release of trapped charge carriers with a microsecond delay [12]. As a result of those correlated breakdowns the charge associated with each detected photon is slightly different, creating an excess noise [13,14].

The output signal of SiPM is composed of pulses generated by triggered SPAD pixels. More or less complex circuit models have been elaborated for studying the output pulse waveforms, for simulations, and for optimizing the readout electronic circuits. The usual approach consists in a combination of linear RC structures, whose complexity depends on the number of parasitic components considered, with controlled switches to model the breakdown [5,15,16]. Although the approach works well for Spice simulations, it is also desirable to express the pulse waveform by means of an analytical function. Many papers, such as [15,17], suggest using the double-exponential waveform for the output pulse approximation. The paper [18] presents a more complex model. Based on the double-exponential approximation of the pixel current, the multiple-exponential approximation of the output pulse is proposed. A similar result based on a detailed analysis of pixel layout can be found in [16]. Depending on the internal chip design, the output pulse may contain a “fast” derivative component as a result of the non-negligible parasitic capacitance of the quenching resistor [19]. The fast component can improve the timing resolution of photon detection [20].

The work presented in this paper was motivated by the problem of detecting very weak periodic optical pulses in the presence of background radiation, which can be found, for example, in LIDAR applications. In this case, the SiPM response is indistinguishable from pulses that are due to the optical background for one shot of the probing laser. Multiple laser pulses should be transmitted, and the response is obtained by means of statistical processing of the SiPM output, a technique known as correlated averaging [21,22].

The output signal of SiPM is a continuous-time stochastic process. The Monte Carlo simulation, used by many authors [23,24,25], is a straightforward solution, which allows including such phenomena as correlated pulses, decreased detection efficiency during pixel recharge, and a finite number of pixels in the matrix. However, Monte Carlo is a numerical method without the possibility of obtaining a closed-form analytical solution with in-depth insight. A series of theoretical papers by S. Vinogradov [26,27] are focused on including the correlated events into the classical Poisson process. The papers are focused on the detection of scintillation events without explicitly describing the output signal. A similar problem is treated in [28], where a detection of a distinguishable scintillation event is studied.

The paper [29] focuses on obtaining a solution for an output pulse rate incorporating non-ideal phenomena of SiPM. The method presented leads to an analytical solution for the stationary case. However, in the non-stationary case, the solution is only available in a discretized form. A comprehensive statistical model for the output charge of SiPM for an arbitrary light-pulse shape is presented in [30], but not for the directly measurable voltage. A simplified solution for the output signal statistics is presented in [31] with a focus on the signal-to-noise ratio of a single event and not the complete waveform.

The presented paper combines the well-established multiple-exponential modeling of SiPM pulses with the statistical theory and derives closed-form expressions for the mean and variance of the stochastic output signal for high-pass coupled SiPMs. To the best knowledge of authors, the topic has not been published yet. It can be shown that the averaged pulse differs from the directly measurable pulses at the SiPM output. The presented model is simple in that it allows an analytical solution, but at the same time, it includes all essential phenomena. The theoretical waveforms can be used to design optimum laser pulse waveforms or advanced filtering in full-wave detection techniques [32]. Section 2 of the paper presents the mathematical procedure, and Section 3 describes the experimental setup and measurement results.

## 2. Modeling of SiPM Signals

### 2.1. Approximation of Output Pulse

**Figure 1:**
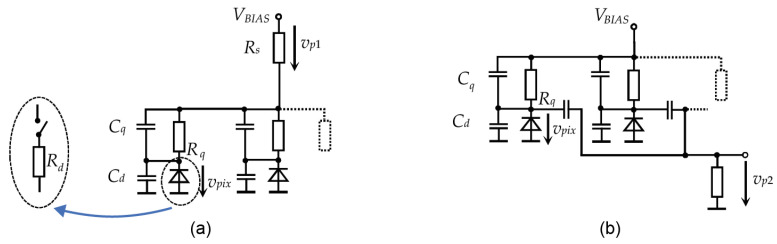
Methods of sensing the output pulses of analog SiPM: (**a**) Measuring the supply current pulses $v_{p1}$ through a small series resistor $R_{s}$ [15]. (**b**) A special output $v_{p2}$ with capacitive coupling [7].

The analog SiPM represents a matrix of single-photon avalanche detectors, often called pixels, with integrated quenching resistors, which are connected to a common supply bus [33]. The output signal is then a superposition of pulses from individual pixels. Figure 1 shows two possible methods of collecting the output signal.

The commonly used principle of obtaining the output signal shown in Figure 1a is based on measuring current pulses on the common supply bus [15]. Note that Figure 1a shows only the principle schematic diagram. A more practical arrangement with a grounded sensing resistor, which can be directly connected to a broadband amplifier, is shown in [34].

**Figure 2:**
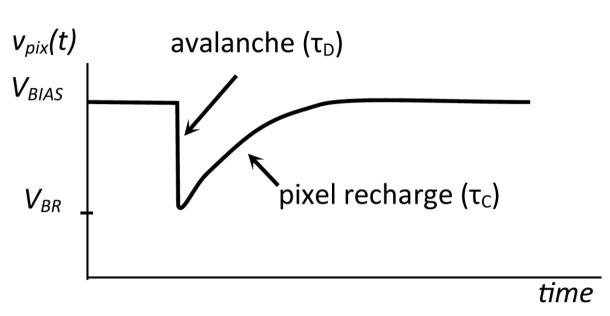
Waveform of the pixel voltage $v_{pix}\left(t\right)$ for a single avalanche event.

APDs in pixels are biased to a voltage $V_{BIAS}$, which is above their reverse breakdown voltage $V_{BR}$, resulting in a Geiger-like behavior [35]. The difference $V_{OV}=V_{BIAS}-V_{BR}$ is called “overvoltage”, and its value determines many parameters of the SiPM [33]. An incident photon triggers, with a certain probability, a self-sustaining avalanche breakdown causing a sub-nanosecond rise in the reverse current. After triggering the avalanche, the photodiode represents a relatively small resistance $R_{d}$ and its reverse voltage decreases rapidly with a time constant $\tau_{D}$. When the voltage reaches approximately the level of $V_{BR}$, the avalanche ceases and the diode becomes non-conducting again. Then, the pixel node capacitance is recharged through the quenching resistor $R_{q}$ with a time constant $\tau_{C}\gg \tau_{D}$, Figure 2.

Based on numerous studies, such as [15,18,36], the standard practice is to approximate the avalanche pulse with the double-exponential waveform. Let us remove, for simplicity, the bias from the $v_{pix}$ waveform and consider the pulse, triggered at t=0, with the positive sign

(1) $$v_{b}\left(t\right)=V_{BIAS}-v_{pix}=V_{0}\;\left(e^{-\alpha t}-e^{-\beta t}\right)\;\sigma \left(t\right),$$

where $\alpha =1/\tau_{C}$, $\beta =1/\tau_{D}$, and σ(t) is the Heaviside step function, which guarantees that $v_{b}\left(t\right)=0$ for t≤0. The parameter $V_{0}$ can be determined from the pulse amplitude.

In the theoretical case of zero parasitic capacitance of $R_{q}$$\left(C_{q}=0\right)$ the supply current pulse as well as the voltage pulse $v_{p1}$ across $R_{s}$ can also be represented by the double-exponential waveform (1). As discussed in [18,19], the nonzero $C_{d}$ causes a “fast” component to appear on the output pulse waveform, which can be modeled using multiple exponential terms because the transfer function from a pixel to the output is more complex

(2) $$v_{p1f}\left(t\right)=\left[V_{1}\left(e^{-\alpha_{1}t}-e^{-\beta_{1}t}\right)+V_{2}\left(e^{-\alpha_{2}t}-e^{-\beta_{2}t}\right)\right]\sigma \left(t\right).$$

**Figure 3:**
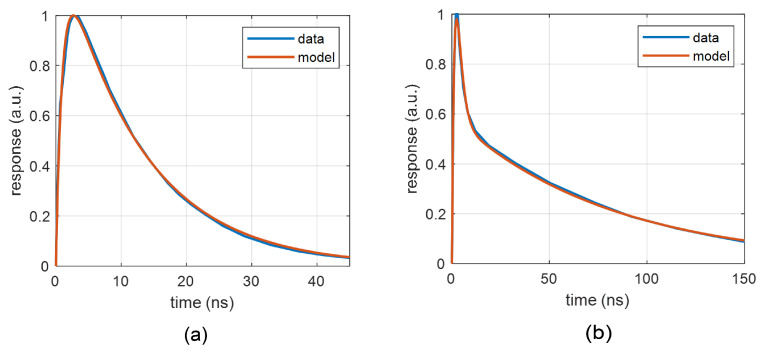
Approximation of the normalized output waveform for ON Semi SiPMs: (**a**) 10 μm-pixel device (MICRORB-10010) with the double-exponential response (1): $V_{0}=1.343$, $\alpha =8.076\times 10^{7}$ s${}^{-1}$, $\beta =1.044\times 10^{9}$ s${}^{-1}$; (**b**) 35 μm-pixel device (MICRORB-10035) with the fast component and multiple-exponential approximation (2): $V_{1}=0.5896$, $\alpha_{1}=1.233\times 10^{7}$ s${}^{-1}$, $\beta_{1}=0.4610\times 10^{9}$ s${}^{-1}$, $V_{2}=11.096$, $\alpha_{2}=0.5069\times 10^{9}$ s${}^{-1}$, $\beta_{2}=0.5914\times 10^{9}$ s${}^{-1}$.

Figure 3 illustrates the use of waveforms (1) and (2) for the normalized responses $v_{p1}$ of two devices from ON Semi, which differ in the fast component. The response of the 10 μm-pixel device can be approximated with the double-exponential function (1). However, the 35 μm-pixel device shows a clear “fast” component in the response, thus the multiple-exponential function (2) should be used. The data for fitting were obtained by means of digitizing reference waveforms from [37], where the device was illuminated with a 50 ps laser pulse at 420 nm wavelength. Note that fitting functions (1) or (2) to the measured response represents an approximation of complex dynamics by a system of the second or the fourth order, respectively. The measurement of nanosecond pulses requires a careful design of laboratory fixtures to avoid wave reflections, which usually appear as overshoots or oscillations on the measured waveforms.

The typical charging times, i.e., the pulse tails, may reach hundreds of nanoseconds. The slow tail can be suppressed by high-pass filtering [3], which can be done externally or by means of a modification of the device design. The SensL Company has introduced a modification of the common SiPM design, adding another terminal with capacitive coupling to individual pixels [7], see Figure 1b.

To get a reasonable, yet simple, approximation of the pulses $v_{p2}$ (Figure 1b), let us start with the transfer function of a simple RC high-pass filter of the first order:

(3) $$H\left(s\right)=\frac{s\tau_{H}}{1+s\tau_{H}},$$

where $\tau_{H}$ is the time constant (or practically the dominant time constant). The transfer function corresponds not only to the device in Figure 1b, but also to all arrangements where a capacitive coupling is used to remove the DC component from the amplified signal.

The corresponding impulse response of the filter (3) will be

(4) $$h\left(t\right)=\delta \left(t\right)-\frac{1}{\tau_{H}}e^{-t/\tau_{H}},$$

where δ(t) is the Dirac pulse. In other words, the filter realizes a lossy derivative of the input signal.

Let us consider the pixel pulse (1). The output voltage is given as a convolution of the pulse with h(t). For the double-exponential waveform and zero initial conditions, we have

(5) $$v_{p2}=h\left(t\right)*v_{b}\left(t\right)=V_{0}\left(Ae^{-\alpha t}+Be^{-\beta t}+Ce^{-\gamma t}\right)\sigma \left(t\right),$$

where “*” denotes the convolution, $A=-\frac{\alpha }{\gamma -\alpha }$, $B=\frac{\beta }{\gamma -\beta }$, $C=\frac{\gamma (\alpha -\beta )}{\left(\gamma -\alpha )(\gamma -\beta \right)}$, and $\gamma =1/\tau_{H}$. It can be easily verified that the filtering removed the DC component

(6) $$\int_{0}^{\infty }v_{p2}\left(t\right)\;dt=0.$$

**Figure 4:**
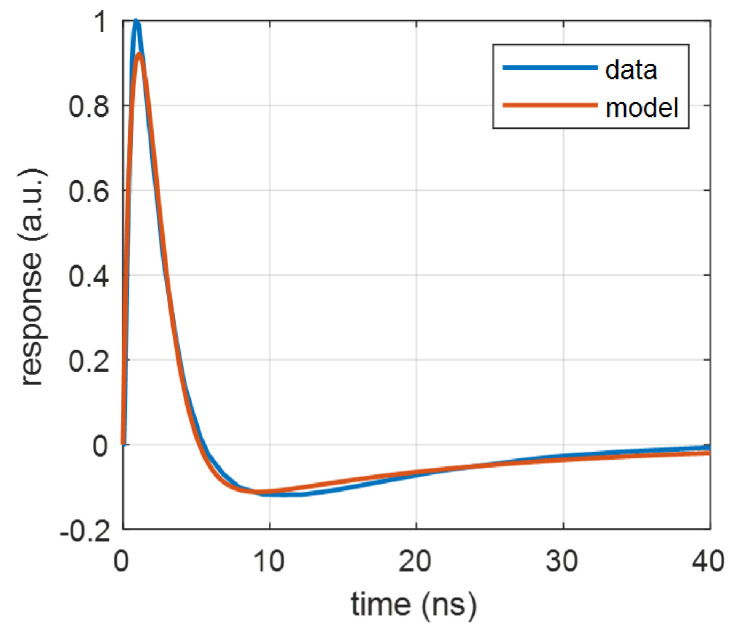
Fitting the function (5) to the measured capacitive-output waveform $v_{p2}$ for MICRORB-10010: $V_{1}=2.315$, $\alpha =5.860\times 10^{7}$ s${}^{-1}$, $\beta =1.045\times 10^{9}$ s${}^{-1}$, $\gamma =0.7067\times 10^{9}$ s${}^{-1}$.

Figure 4 shows fitting the approximation (5) to the output waveform $v_{p2}$ of a 10 μm-pixel device ON Semi MICRORB-10010 (Phoenix, AZ, USA). The maximum approximation error at the peak is 8%. Note that the identified parameters α and β of (5), see Figure 4, differ from those shown in Figure 3 for the same device. The measured waveform $v_{p1}$ on the main output differs from the pixel voltage $v_{pix}$, and therefore $v_{p2}$ is not a simple high-pass version of $v_{p1}$. Similar to Figure 3, the reference waveforms were taken from [37] for the device illuminated by a 50 ps laser pulse.

### 2.2. Stochastic Process of Photon Detection

The optical power illuminating the detector active area can be considered as the Poisson flow of photons, which trigger, with a certain probability, avalanche breakdowns in the pixel matrix. If there are enough other pixels in the fully charged state during the recovery period of a triggered pixel, the event rate depends linearly on the rate of incident photons, i.e., the photomultiplier operates in the linear regime [38].

As the rate of incident photons increases, fewer and fewer cells are in the fully charged state and the probability of photon detection decreases. The device becomes saturated. In case of short light pulses $\left(t_{pulse}\ll \tau_{C}\right)$, the saturation process can be modeled by using the formula [38]

(7) $$N_{t}=N_{pix}\left(1-e^{\frac{-PDE\;N_{ph}}{N_{pix}}}\right),$$

where $N_{t}$ is the number of triggered pixels, $N_{pix}$ is the total number of pixels, $N_{ph}$ is the number of incident photons, and PDE is the photon detection efficiency, i.e., the probability that an incident photon triggers the avalanche. For $\left(PDE\;N_{ph}\right)\ll N_{pix}$, we obtain an obvious relation

(8) $$\lambda_{t}=\lambda_{ph}PDE,$$

where $\lambda_{ph}$ is the average photon rate, and $\lambda_{t}$ is the average rate of photon-induced avalanche pulses in the SiPM device. There are several physical mechanisms that cause a primary avalanche event to trigger, with a certain probability and delay, avalanches in the same or neighboring pixels [14,39]. The afterpulsing and crosstalk causes the number of detected pulses to be higher than the number of primary photo-events.

To obtain representative results, let us assume that the photomultiplier operates in the linear regime and the process of avalanche breakdowns can be modeled as a non-stationary (or non-homogeneous) Poisson process [40] whose only parameter is the time-variable average event rate λ(t)

(9) $$\lambda \left(t\right)=\lambda_{0}+\lambda_{p}\left(t\right).$$

The constant part $\lambda_{0}$ corresponds to the spontaneous breakdowns (Dark Count Rate) and the constant background radiation. The time-dependent part $\lambda_{p}\left(t\right)=K\;p_{R}\left(t\right)$ corresponds to the received optical pulse $p_{R}\left(t\right)$. *K* is the ratio of proportionality depending on detector operating conditions and the photon wavelength.

**Figure 5:**
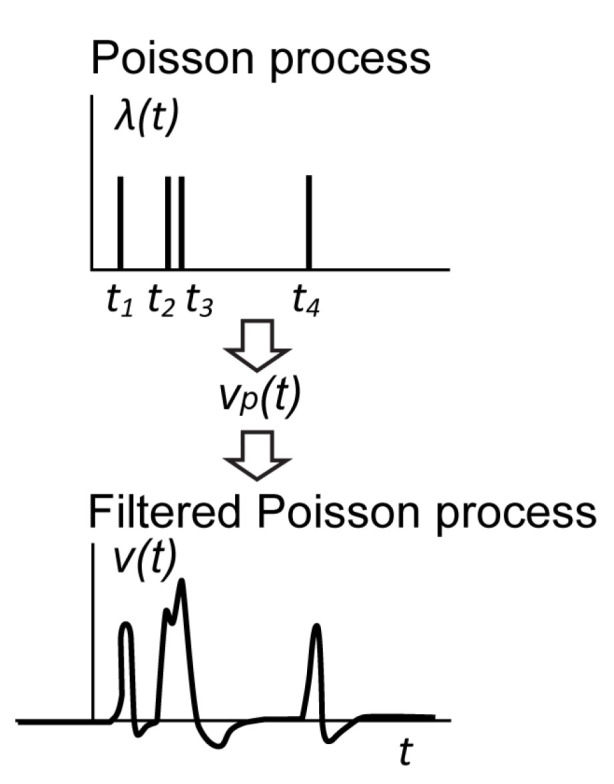
Output signal of SiPM as a filtered Poisson process.

Let us consider, for simplicity, that each avalanche breakdown results in the same output pulse. The output voltage v(t) is a superposition of the pulses shifted in time. Note that if two or more events are close enough, the pulses cannot be separated, as shown in Figure 5 for the events $t_{2}$ and $t_{3}$.

**Figure 6:**
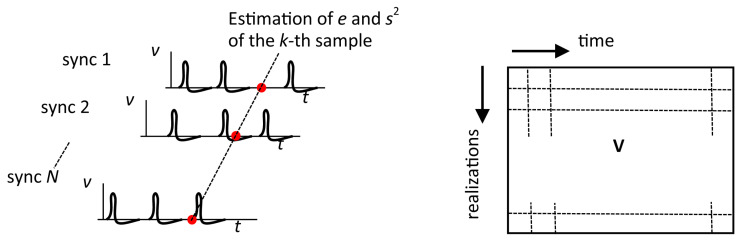
Multiple realizations of the process v(t) and the matrix of samples.

The output signal v(t) is a filtered non-stationary Poisson process. Random Dirac pulses of the Poisson process corresponding to the avalanche events excite a hypothetical linear dynamical system with the causal impulse response $v_{p}\left(t\right)$. The linear system represents the SiPM itself and all the other analog blocks (preamplifier, filter, etc.) in the acquisition chain. The output voltage v(t) is a continuous-time non-stationary stochastic process with the mean and variance [40]

(10) $$E\left[v\left(t\right)\right]=e\left(t\right)=\lambda \left(t\right)*v_{p}\left(t\right),$$

(11) $$VAR\left[v\left(t\right)\right]=s^{2}\left(t\right)=\lambda \left(t\right)*v_{p}^{2}\left(t\right),$$

where “*” denotes the convolution, and $v_{p}\left(t\right)$ is the pulse waveform, which can be either (2) or (5). However, the subsequent analyses will be performed for the high-pass filtered waveform (5), for which the experimental verification was performed in Section 3. Note that the characteristics (10) and (11) can only be estimated from multiple realizations of the stochastic process v(t), but not from the time series of one realization. Therefore, it is applicable in situations where the process can be periodically restarted, such as ranging applications or probing specimens with periodic laser pulses. It is expected that the pulse period is long enough for all transients to decay. The sampling of v(t) in the receiver is started by the synchronization pulse from the transmitter. The pulse transmission repeats and we have multiple realizations, i.e., multiple sampled vectors, of the non-stationary process in the memory, as shown in Figure 6. Then, the statistical parameters of v(t) can be estimated from the corresponding samples across the realizations.

Having the matrix $V\in R^{N\times M}$ of *M* samples of output voltage for each of the *N* realizations, the mean and variance of the *k*-th sample can be estimated in a standard way:

(12) $$e_{k}=\frac{1}{N}\sum_{i=1}^{N}V_{i,k},$$

(13) $$s_{k}^{2}=\frac{1}{N-1}\sum_{i=1}^{N}{\left(V_{i,k}-e_{k}\right)}^{2}.$$

### 2.3. Statistical Analysis

**Figure 7:**
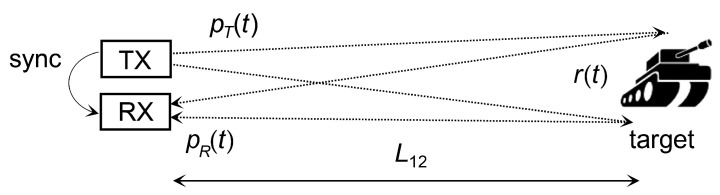
Rangefinder with a complex target.

The reception of low photon-count signals whose power is below the background radiation is typical, for example, for ranging applications. In this case, the SiPM output pulses during the reception of the reflected optical signal are indistinguishable from background pulses for one particular realization of v(t). The signal detection is possible only by estimating (10) from multiple laser shots, which is known as the technique of correlated averaging. Figure 7 shows the configuration of the range-finding system considered. However, the methodology is applicable to all setups with a nontrivial optical signal path.

Let a single transmitted pulse with the power waveform $p_{T}\left(t\right)$ be reflected back by the target and received as $p_{R}\left(t\right)$. The target surface may be complex and the received pulse will include components of $p_{T}\left(t\right)$ with a different delay (for example, a one-meter depth corresponds to a 6.6 ns delay). Therefore, the target should be characterized by its impulse response r(t) [41]. If we omit the propagation attenuation and the propagation delay $2L_{12}/c$, the received pulse waveform will be

(14) $$p_{R}\left(t\right)=p_{T}\left(t\right)*r\left(t\right).$$

If the target is a perpendicular plane at a sufficient distance, the impulse response can be approximated by the Dirac pulse [41].

Let us consider the reflected pulse reception with non-negligible background radiation. Because the sum of Poisson processes is also a Poisson process [40], the components of (9) can be analyzed independently. The rate $\lambda_{0}$ corresponds to the stationary process of the background radiation and dark pulses, and $\lambda_{p}\left(t\right)$ corresponds to the non-stationary reception of the optical signal $p_{R}\left(t\right)$.

Let us assume an analysis of the high-pass filtered signal (5). Combining (9) with (10) and (11), we have for the mean and variance

(15) $$e\left(t\right)=\left(\lambda_{0}+\lambda_{p}\left(t\right)\right)*v_{p2}\left(t\right)=e_{0}\left(t\right)+e_{p}\left(t\right),$$

(16) $$s^{2}\left(t\right)=\left(\lambda_{0}+\lambda_{p}\left(t\right)\right)*v_{p2}^{2}\left(t\right)=s_{0}^{2}\left(t\right)+s_{p}^{2}\left(t\right).$$

Let us suppose that the analog processing channel including the photomultiplier has been switched on sufficiently long before the sampling starts. In the limit case, we have for the stationary part

(17) $$\lim_{t\to \infty }e_{0}\left(t\right)=\lim_{t\to \infty }\lambda_{0}*v_{p2}\left(t\right)=\lambda_{0}\lim_{t\to \infty }\int_{0}^{t}v_{p2}\left(\tau \right)d\tau =0,$$

(18) $$\lim_{t\to \infty }s_{0}^{2}\left(t\right)=\lim_{t\to \infty }\lambda_{0}*v_{p2}^{2}\left(t\right)=\lambda_{0}\lim_{t\to \infty }\int_{0}^{t}v_{p2}^{2}\left(\tau \right)d\tau =S_{0}^{2}.$$

Because $v_{p2}$ does not have a DC component (see (6)), the mean corresponding to the background radiation is always zero. It can be easily shown that (18) converges to

(19) $$S_{0}^{2}=\lambda_{0}V_{0}^{2}\frac{{\left(\beta -\alpha \right)}^{2}}{2(\alpha +\gamma )(\beta +\gamma )(\alpha +\beta )},$$

which always gives a positive value, confirming the contribution of the background radiation to the detected noise.

Let us consider an ideal square laser pulse and an ideal target with r(t)=δ(t). Without the propagation delay, the non-stationary received component will be

(20) $$\lambda_{p}\left(t\right)=\Lambda_{0}\left(\sigma \left(t\right)-\sigma \left(t-T_{w}\right)\right),$$

where $\Lambda_{0}$ is the mean photon rate of the pulse, and $T_{w}$ is the pulse width. The corresponding mean and variance will be after some rearrangements

(21) $$e_{p}\left(t\right)=\lambda_{p}\left(t\right)*v_{p2}\left(t\right)=\Lambda_{0}\left(\int_{0}^{t}v_{p2}\left(\tau \right)d\tau -\int_{0}^{t}v_{p2}\left(\tau -T_{w}\right)d\tau \right),$$

(22) $$s_{p}^{2}\left(t\right)=\lambda_{p}\left(t\right)*v_{p2}^{2}\left(t\right)=\Lambda_{0}\left(\int_{0}^{t}v_{p2}^{2}\left(\tau \right)d\tau -\int_{0}^{t}v_{p2}^{2}\left(\tau -T_{w}\right)d\tau \right).$$

Note that $v_{p2}$ is causal, and therefore $v_{p2}\left(t\right)=0$ for t<0 in (21) and (22).

**Figure 8:**
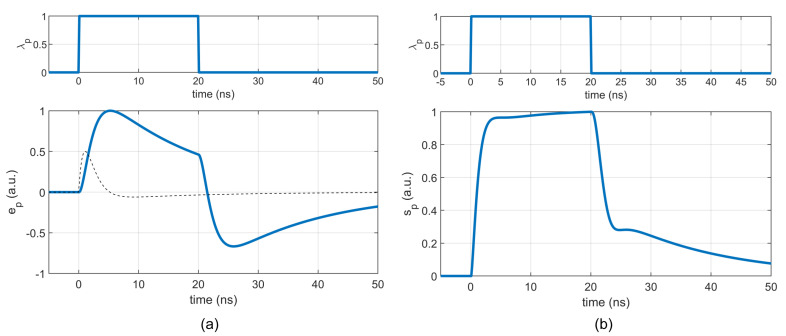
Mean and standard deviation of v(t) for $T_{w}=20$ ns, and $\Lambda_{0}=1$: (**a**) Normalized mean $e_{p}\left(t\right)$ computed using (21). The dashed line shows a single pulse of $v_{p2}$ for comparison. (**b**) Normalized standard deviation $s_{p}\left(t\right)$ computed using (22).

Figure 8 shows the simulated response of the mean $e_{p}\left(t\right)$ and standard deviation $s_{p}\left(t\right)$ to a 20 ns square optical pulse for the waveform (5) with parameters from Figure 4. To better understand the waveform, the plot also contains a single pulse $v_{p2}\left(t\right)$ plotted with a dashed line. With respect to (21), $de_{p}\left(t\right)/dt$ ∝ $v_{p2}\left(t\right)$ for $0<t<T_{w}$. Thus, the maximum edge slope of $e_{p}\left(t\right)$ corresponds to the maximum of $v_{p2}\left(t\right)$ and the maximum of $e_{p}\left(t\right)$ corresponds to the zero crossing of $v_{p2}\left(t\right)$.

## 3. Experimental Study

### 3.1. Test Setup

**Figure 9:**
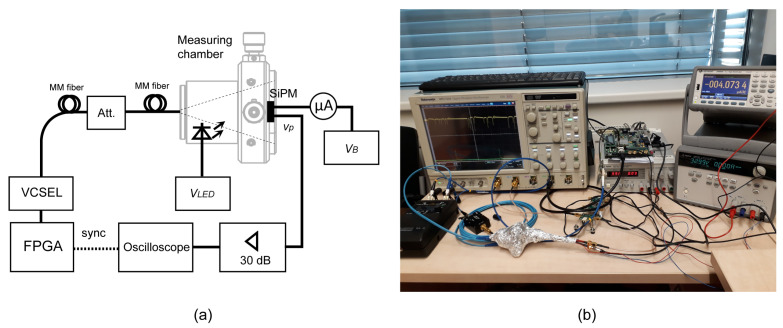
Block diagram (**a**) and photograph (**b**) of the experimental setup. The measuring chamber is wrapped in aluminum foil to block the ambient light.

Figure 9 shows the block diagram and photograph of the experimental setup for measurements of SiPM response to weak optical pulses. The photomultiplier is located in a chamber and is illuminated by a multimode optical fiber. The optical pulses are generated by an 850 nm VCSEL from a modified Gigabit Ethernet SFP module. The laser is driven directly from a port of Xilinx ML-505 FPGA board (San Jose, CA, USA) without any DC pre-bias. A RC network is used between the 3.3 V FPGA output and the laser to limit the operating current and to compensate for the slow rising edge of unbiased VCSEL. This arrangement ensures that the laser is actually turned off outside the pulse duration. An optical attenuator decreases the pulse power to a suitable level. The chamber also contains a regulated and optically attenuated LED to simulate the background radiation. The SiPM supply current is monitored with a precise Keysight 34465A ammeter (Colorado Springs, CO, USA).

The SiPM output is connected to a 30 dB wideband preamplifier based on INA-02184 gain block (Gain = 30 dB, bandwidth 0.1 MHz–2 GHz). The amplified signal from SiPM and synchronization pulses from FPGA are digitized using Tektronix DPO 7254 oscilloscope (Beaverton, OR, USA). The instrument is capable of two-channel recording with the length of $25\times 10^{6}$ samples at a speed of 5 GSa/s. Complete data processing is done in Mathworks Matlab (Natick, MA, USA).

**Figure 10:**
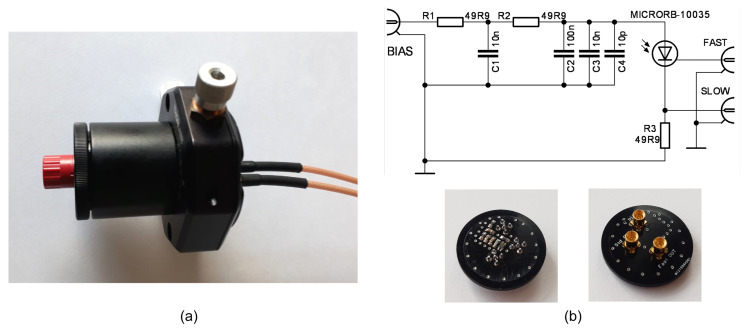
Measuring chamber (**a**) and the SiPM fixture (**b**).

Figure 10 shows the measuring chamber designed from Thorlabs optomechanical parts. The ON Semi photomultiplier MICRORB-10035 (Phoenix, AZ, USA) is placed in the middle of a 1 inch round-shape printed circuit board with MMCX connectors. The biasing scheme is recommended by the manufacturer [34]. The positive terminal of the power supply is grounded to get ground-referenced output signals. The fixture has two outputs: “SLOW” ($v_{p1}$) sensing the photomultiplier supply current and “FAST” ($v_{p2}$) with a capacitive-coupled output shown in Figure 1b. Table 1 summarizes the typical datasheet parameters of MICRORB-10035. All measurements were done for bias voltage $V_{BIAS}$ = 33 V. During the experiments, the chamber was wrapped in aluminum foil to block room light, which leaked in through the printed circuit board, as shown in Figure 9b.

### 3.2. Event Rate and DC Current

**Figure 11:**
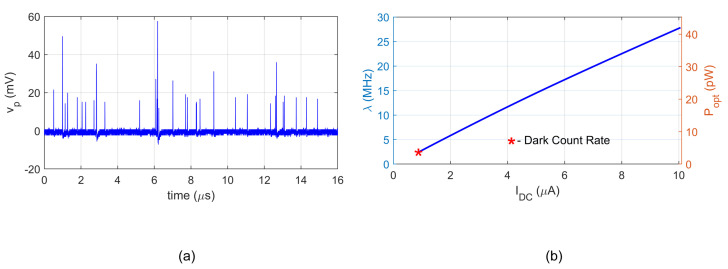
Measuring the event rate: (**a**) Example record with multi-event pulses. (**b**) Estimated event rate as a function of DC current.

During the experiment, the SiPM active area was illuminated by a constant optical power from the LED embedded in the measuring chamber. The VCSEL pulse generator was switched off. The DC current of SiPM is composed of charges drawn by each avalanche breakdown. With respect to a relatively low afterpulse rate (see Table 1), we can consider that each breakdown represents approximately the same charge. However, the breakdowns can occur almost simultaneously resulting in a higher pulse. Figure 11 shows an example record with different amplitudes.

As this study is focused on the detection of low-photon-count pulses, the measurement was performed for low event rates, where the individual pulses are distinctive in the output waveform. A procedure written in Matlab was used to detect single, double, and triple events to correctly estimate the rate. The result in Figure 11b was obtained from a single-channel record of $50\times 10^{6}$ samples at a rate of 5 GSa/s.

The first point in Figure 11b corresponds to the room-temperature dark count rate. The obtained values DCR = 2.5 MHz and $I_{dark}\;=\;0.83\;\mu$A correspond well to datasheet values. Then, the LED current was increased and the process of spontaneous “dark” breakdowns merged with photon-induced breakdowns with an expected linear dependence of the rate on current

(23) $$\lambda \approx K\;I_{DC},$$

where the proportionality constant was found *K* = 2.77 MHz/μA. The right vertical axis of Figure 11b shows the corresponding average optical power computed from the photomultiplier responsivity.

### 3.3. Pulse Analysis

The FPGA board generates optical pulses of selectable length with a repetition frequency of 2 MHz. The oscilloscope records SiPM output signal and synchronization pulses from FPGA, both of which are used by software to find the (re)start of the stochastic process. The repetition rate was chosen as a compromise between a sufficient time for transient decay and the number of events that can be recorded in the oscilloscope memory.

**Figure 12:**
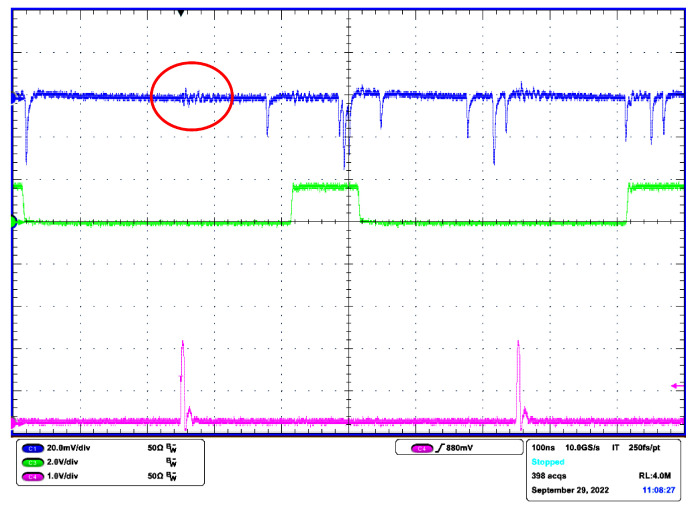
Raw signals captured by oscilloscope (Channel 1—SiPM output $v_{p2}$; Channel 3—FPGA pulse to VCSEL, Channel 4—sync pulses). The red circle shows electrical crosstalk from sync pulses.

Figure 12 shows an example record of raw signals for the background rate $\lambda_{0}$ = 5.54 MHz ($I_{DC}$ = 2 μA) and pulse rate $\Lambda_{0}$ = 27.7 MHz (see (9) and (20)). The rates we set by means of measuring the DC current using relation (23). The measuring of DC current is much faster during experiments than determining event rates in the software. The FPGA pulse length was set to 100 ns with 2 ns rising and falling times. The pulse is placed between the syncs so that the measurement is not affected by the electrical crosstalk, as shown in Figure 12. Note that the $v_{p2}$ pulses are actually negative and they are inverted for Matlab postprocessing. The average number of pulse events per pulse duration is 3.32 including the background radiation. The waveform of $v_{p2}$ documents that during the optical pulse reception, only a few avalanche events occurred. Given the average event rate λ, the probability that at least one event occurs in the interval *T* is

(24) $$P\left(k>0,T\right)=1-e^{-\lambda T}.$$

**Figure 13:**
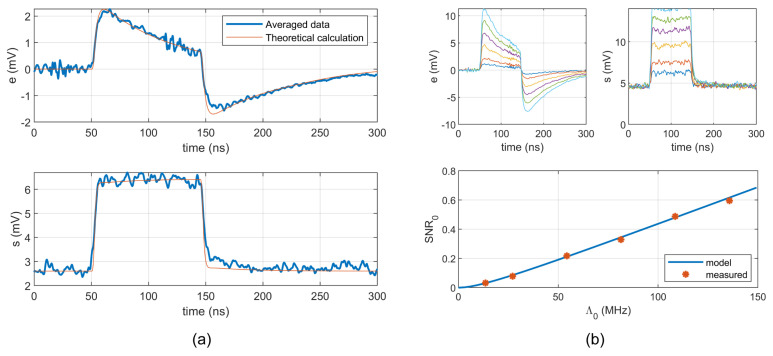
Statistical processing of recorded signals: (**a**) Estimation of e(t) and s(t) for $\lambda_{0}$ = 5.54 MHz and $\Lambda_{0}$ = 27.7 MHz computed from 9998 records. (**b**) Estimation of $SNR_{0}$ for different pulse amplitudes.

The estimations of mean and standard deviation of the non-stationary stochastic process were computed from *N* = 9998 recorded repetitions, i.e., from 9998 realizations of the process, using (12) and (13). The captured time interval was 5 ms with $25\times 10^{6}$ samples. The pulse origin was shifted to 50 ns in Figure 13a, which shows the estimated waveforms of e(t) and s(t). The mean of the stationary background process is zero. Therefore, the waveform e(t) corresponds directly to (21). However, the standard deviation of the background process $S_{0}$ is not zero and therefore sums with the pulse deviation $s_{p}\left(t\right)$ (22).

(25) $$s^{2}\left(t\right)=S_{0}^{2}+s_{p}^{2}\left(t\right)$$

as both processes are inseparable. Note that the estimation of e(t), i.e., the correlated averaging, is a stochastic process itself, as it is computed from a finite number of repetitions. The standard deviation of the estimated mean will be

(26) $$s_{\Sigma }\left(t\right)=s\left(t\right)/\sqrt{N}.$$

A software procedure was used to find “unity” pulses in the recorded signal. The waveform (5) was fitted to the pulses with following parameters: $V_{0}$ = 22.5 mV, $\alpha \;=\;1.51\times 10^{7}$ s${}^{-1},$$\beta \;=\;1.98\times 10^{8}$ s${}^{-1},$ and $\gamma \;=\;3.76\times 10^{8}$ s${}^{-1}$. The theoretical waveforms for *e* and *s* were calculated using (21) and (22), and plotted in the same figure for comparison.

The computed estimation of e(t) may be used in the detection of the pulse arrival time. Let us consider a simple algorithm searching for the peak of e(t) occurring at $t_{peak}$. In this case, we can define the signal-to-noise ratio as

(27) $$SNR_{peak}=e{\left(t_{peak}\right)}^{2}/s_{\Sigma }^{2}\left(t_{peak}\right).$$

As the integral (18) converges quickly after the pulse has started, we can use (19) for the denominator of (27)

(28) $$s_{\Sigma }^{2}\left(t_{peak}\right)\approx \frac{\lambda_{0}+\Lambda_{0}}{N}\frac{V_{0}^{2}{\left(\beta -\alpha \right)}^{2}}{2(\alpha +\gamma )(\beta +\gamma )(\alpha +\beta )}.$$

The peak value of e(t) is then

(29) $$e\left(t_{peak}\right)=\Lambda_{0}V_{0}\max\left(\int_{0}^{t}Ae^{-\alpha \tau }+Be^{-\beta \tau }+Ce^{-\gamma \tau }d\tau \right).$$

Finally, (27) can be expressed using (28) and (29) as

(30) $$SNR_{peak}\approx N\frac{\Lambda_{0}^{2}}{\lambda_{0}+\Lambda_{0}}S\left(\alpha ,\beta ,\gamma \right)=N\;SNR_{0}.$$

The first term of (30) depends on the event rates and the number of repetitions, while the second term only depends on the single avalanche pulse approximation (5). For the tested setup, the constant was found to be $S\left(\alpha ,\beta ,\gamma \right)=5.22\times 10^{-9}$. The resulting ratio depends linearly on *N* with a proportionality coefficient $SNR_{0}$.

In the second experiment, the response of SiPM was measured for the background rate $\lambda_{0}$ = 16.6 MHz ($I_{DC}$ = 6 μA) with amplitudes $\Lambda_{0}$ ranging from 13.8 MHz to 138 MHz ($I_{DCtotal}$ = 7 μA to 16 μA). Figure 13b shows the predicted and measured parameter $SNR_{0}$ from (30).

## 4. Discussion

The presented statistical model is valid on the assumption of the validity of the linear response of the SiPM to the sum of Poisson processes at the input, i.e., assuming the validity of (8) as a local approximation of (7). Thus, the linear model is valid for the reception of very weak signals for which the saturation phenomena will not appear.

The used double-exponential approximation for SiPM pulses represents a good compromise between simplicity and accuracy. The function admits an analytical solution to the mean and the standard deviation of the stochastic process associated with SiPM operation. This gives the possibility of obtaining analytical formulae simple enough to provide insight into the detection process. In addition, the theoretical waveforms can be used to design matched filters or pulse detection techniques, e.g., in ranging and LIDARs. It is possible to formulate the following partial conclusions based on the theoretical waveforms:

- The rise time of $e_{p}\left(t\right)$ is longer than the rise time of $v_{p2}\left(t\right)$ even for an ideal square optical pulse, which may affect the accuracy of pulse arrival time estimation. In fact, the waveform for $0<t<T_{w}$ is given by the first integral in (21). Therefore, the estimated $e_{p}\left(t\right)$ corresponds to the original waveform (1) since $v_{vp2}\left(t\right)$ is its lossy derivative.
- As a consequence of (6), $e_{p}\left(t\right)$ starts to decrease after reaching its maximum. Therefore, there exists some optimum pulse width $T_{w}$, above which the amplitude of $e_{p}\left(t\right)$ does not grow.

The presented model is based on a simple Poisson process, neglecting the correlated pulses due to crosstalk. It can be seen on the falling edge of both waveforms in Figure 13a, where the theoretical waveform deviates slightly from experimental results. The future research will be aimed at the inclusions of correlated events.

## Figures and Tables

**Table 1:** Main parameters of MICRORB-10035.

Parameter	Typical
Breakdown Voltage $\left(V_{BR}\right)$	25 V
Overvoltage $\left(V_{OV}\right)$	7 V (10 V max)
Number of Microcells	620
Responsivity (905 nm, typ. $V_{OV}$)	240 kA/W
Dark Count Rate	2.6 MHz
Dark Current	1.5 μA
Afterpulsing	1%

## Data Availability

Not applicable.

## Abbreviations

The following abbreviations are used in this manuscript:

APD	Avalanche photodiode
SPAD	Single-photon avalanche detector
SiPM	Silicon photomultiplier
LIDAR	Light detection and ranging
PDE	Photon detection efficiency
DCR	Dark count rate
FPGA	Field programmable gate array
VCSEL	Vertical-cavity surface-emitting laser
LED	Light-emitting diode
